# Etymologia: Markov Chain Monte Carlo

**DOI:** 10.3201/eid2512.ET2512

**Published:** 2019-12

**Authors:** Ronnie Henry

**Keywords:** etymologia, Markov chain Monte Carlo, MCMC, stimulation, posterior distribution, modeling, probabilities, random sampling, Stanislaw Ulam, Andrey Markov

## Markov Chain Monte Carlo

A Markov chain Monte Carlo (MCMC) simulation is a method of estimating an unknown probability distribution for the outcome of a complex process (a posterior distribution). Prior (capturing the concept *prior* to seeing any data) distributions are used to simulate sampling from variables that have known or closely approximated distributions in the complex process. Thus, the prior distributions are known probability distributions that represent uncertainty about a particular attribute of a population prior to data sampling, and the posterior distribution represents estimated uncertainty about a population attribute after data sampling and is conditional on the observed data. 

Monte Carlo (named for the casino in Monaco) methods estimate a distribution by random sampling. Many samples of the prior distributions must be obtained (e.g., many rolls of the dice) to obtain a stable and accurate posterior distribution. The modern version of the Monte Carlo was invented by Stanislaw Ulam and developed early on by John von Neumann and Nicholas Metropolis, the latter of whom suggested the name, as part of follow, on work to the Manhattan Project. Ulam was trying to calculate the probability of laying out a winning game of solitaire from a shuffled deck of 52 cards. Because of the complexity of the calculations, he decided it would be easier to play 100 games of solitaire and count the percentage that won. 

In a Markov chain (named for Russian mathematician Andrey Markov [Figure]), the probability of the next computed estimated outcome depends only on the current estimate and not on prior estimates. For example, if you shuffle a deck of cards 3 times, the outcome of the third shuffle depends only on the state of the cards at the second shuffle, not at the first shuffle. Markov chain Monte Carlo simulations allow researchers to approximate posterior distributions that cannot be directly calculated.

**Figure:**
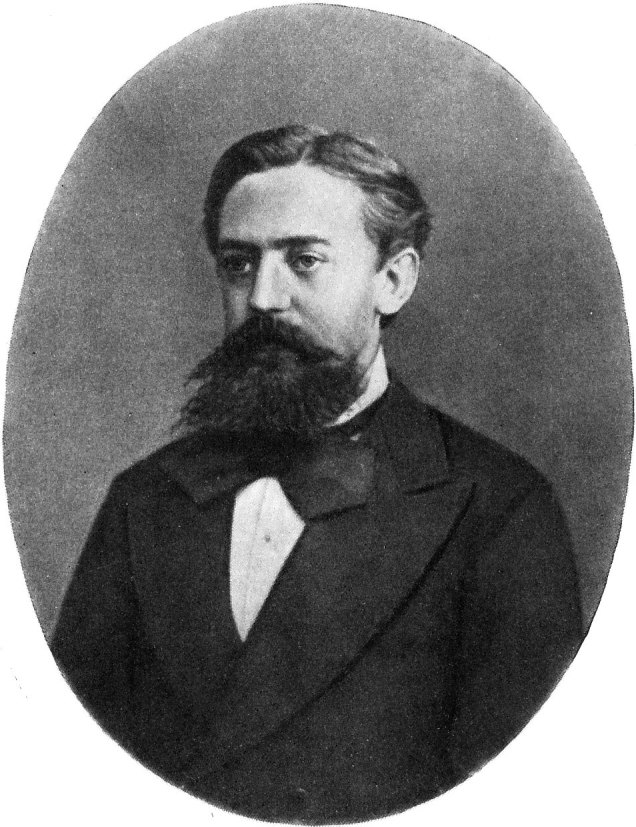
Andrey Markov (1856–1922), photographer unknown, public domain, https://commons.wikimedia.org/w/index.php?curid=1332494
